# Reverse Genetics of RNA Viruses: ISA-Based Approach to Control Viral Population Diversity without Modifying Virus Phenotype

**DOI:** 10.3390/v11070666

**Published:** 2019-07-20

**Authors:** Jean-Sélim Driouich, Gregory Moureau, Xavier de Lamballerie, Antoine Nougairède

**Affiliations:** Unité des Virus Émergents (UVE: Aix-Marseille Univ-IRD 190-Inserm 1207-IHU Méditerranée Infection), 13005 Marseille, France

**Keywords:** reverse genetics systems, RNA virus, ISA, infectious subgenomic amplicons, infectious clone, tick-borne encephalitis virus

## Abstract

Reverse genetic systems are essential for the study of RNA viruses. Infectious clones remain the most widely used systems to manipulate viral genomes. Recently, a new PCR-based method called ISA (infectious subgenomic amplicons) has been developed. This approach has resulted in greater genetic diversity of the viral populations than that observed using infectious clone technology. However, for some studies, generation of clonal viral populations is necessary. In this study, we used the tick-borne encephalitis virus as model to demonstrate that utilization of a very high-fidelity, DNA-dependent DNA polymerase during the PCR step of the ISA procedure gives the possibility to reduce the genetic diversity of viral populations. We also concluded that the fidelity of the polymerase is not the only factor influencing this diversity. Studying the impact of genotype modification on virus phenotype is a crucial step for the development of reverse genetic methods. Here, we also demonstrated that the utilization of different PCR polymerases did not affect the phenotype (replicative fitness in cellulo and virulence in vivo) compared to the initial ISA procedure and the use of an infectious clone. In conclusion, we provide here an approach to control the genetic diversity of RNA viruses without modifying their phenotype.

## 1. Introduction

Reverse genetic methods that enable generation of infectious viruses from DNA copies of their genomes have completely transformed the study of RNA viruses [1,2,3]. Indeed, reverse genetic systems provide opportunities to better understand RNA virus life cycles or mechanisms of pathogenesis, and have contributed to the development of antiviral therapeutics and vaccines [4,5].

Even if the infectious clone (IC) technology remains the most widely used reverse genetic system, this tool remains difficult to employ, particularly because of the toxicity and instability of certain viral sequences expressed in bacteria. Therefore, several other bacterium-free reverse genetic tools have been developed to produce wild type and genetically modified viruses [1]. Among these, the versatile and simple reverse genetic method named ISA (infectious subgenomic amplicons) enables rescue of infectious RNA viruses without requiring an in vitro RNA transcription step, cloning, or propagation of cDNA into bacteria [6]. However, we previously demonstrated with the Chikungunya virus that this method resulted in greater genetic diversity of the viral populations, making this approach unsuitable for mutagenesis experiments that require clonal populations of viruses [7].

Thermostable DNA-dependent DNA polymerases are well known to induce levels of molecular heterogeneity that vary according to their relative specific error rates [8,9,10,11,12]. Therefore, we hypothesized that the artificial viral heterogeneity generated during the PCR step of the ISA procedure (i.e., amplification of several overlapping subgenomic amplicons that encompass the complete viral genome) affects the genetic diversity of viral populations. For this purpose, we previously developed a new ISA-derived method named SuPReMe (subgenomic plasmids recombination method), which is based on the use of subgenomic DNA fragments derived from digested plasmids instead of amplicons. This approach combines the simplicity of the ISA method and its ability to produce a quasi-clonal viral population [7].

Based on these results, we then hypothesized that the use of very high-fidelity PCR polymerases will also reduce the genetic heterogeneity of the subgenomic amplicons, and, consequently, decrease the genetic diversity of the viral populations produced. For this purpose, we used here the tick-borne encephalitis virus (TBEV) as a model. TBEV (family *Flaviviridae*; genus *Flavivirus*; a small enveloped, single-strand positive-sense RNA virus) is a human pathogen, transmitted by ticks of the *Ixodes* genus, responsible for febrile illness and encephalitis in forested regions of Europe and Asia [13,14]. Despite the availability of several vaccines, the incidence of TBEV is increasing across Central and Eastern Europe [15]. In this study, we explored the impact of the fidelity of the PCR polymerase used during the ISA procedure on the genetic diversity of viral populations.

Since the mutant spectrum of viral populations can shape virus phenotype, it is important to study the impact of new reverse genetic approaches on viral phenotype [16]. We previously investigated the impact of using the ISA method on viral phenotype in comparison with using an infectious clone, and we did not observe any difference of replicative fitness in cellulo or of vector competence in *Aedes* mosquitoes [7,17]. In this study, we have also explored the impact of the fidelity of the PCR polymerase used during the ISA procedure on replicative fitness in cellulo, and on virulence, using a mouse model previously described [18,19,20].

## 2. Materials and Methods 

### 2.1. Cells and Animals

Vero cells (derived from an African green monkey kidney; ATCC number CCL-81) were grown at 37 °C with 5% CO_2_ in a minimal essential medium (Thermo Scientific, Waltham, MA, USA) with 7% heat-inactivated fetal bovine serum (FBS; Thermo Scientific), 1% penicillin/streptomycin (PS; 5000 U mL^−1^ and 5000 µg mL^−1^; Thermo Scientific), and 1% glutamine (Gln; 200 mmol L^−1^; Thermo Scientific). HEK-293 cells were grown at 37 °C with 5% CO_2_ in the same medium as the Vero cells, supplemented with 1% non-essential amino acids (Thermo Scientific).

Three week old C57Bl/6J female mice were provided by Charles River laboratories.

### 2.2. Ethics Statement

Animal protocols were reviewed and approved by the ethics committee “Comité d’éthique en expérimentation animale de Marseille—C2EA—14” (protocol number 2504, 03 February 2016). All animal experiments were performed accordingly with the French national guidelines and the European legislation covering the use of animals for scientific purposes.

### 2.3. TBEV Infectious Clone (IC)

A previously described IC of the Oshima 5–10 strain was used in this study [19,21].

The complete viral genome is flanked at the 5′ and 3′ termini, respectively, by the human cytomegalovirus promoter (pCMV) and the hepatitis delta ribozyme followed by the simian virus 40 polyadenylation signal (HDR/SV40pA). 

### 2.4. Preparation of DNA Fragments for the ISA Procedure

The complete viral genome, flanked respectively at the 5′ and 3′ extremities by the pCMV and the cHDR/SV40pA, was amplified by PCR in three overlapping DNA fragments of 4.8 kb, 4.1 kb, and 3.4 kb, as previously described [6]. The IC was used as the template for all PCR amplifications. Primer sequences are listed in Table S1.

Three kits, with different fidelities, were used to perform PCR amplification. Firstly, amplicons were produced using the Platinum PCR SuperMix High Fidelity kit (Thermo Scientific; 3.67 × 10^−6^ errors per nt per cycle; manufacturer’s data). Secondly, they were produced using the Pfu DNA Polymerase, recombinant kit (Thermo Scientific; 2.6 × 10^−6^ errors per nt per cycle; manufacturer’s data). Thirdly, they were produced using the Phusion High Fidelity DNA polymerase kit (Thermo Scientific; 4.4 × 10^−7^ errors per nt per cycle; manufacturer’s data).

For each PCR reaction, final primer concentration was 200 nM and the final volume was 50 µL, including 2 µL of the DNA template (1 ng/µL). The PCR reaction was supplemented with (i) 45 µl of SuperMix (Platinum PCR SuperMix High Fidelity kit), (ii) 5 µL of 10× Pfu buffer, 0.75 µL of Pfu DNA polymerase and 5 µL of dNTP Mix (Pfu DNA Polymerase, recombinant kit), or (iii) 10 µL of 5× Phusion buffer, 0.5 µL of Phusion DNA polymerase, and 1 µL of dNTP Mix (Phusion High Fidelity DNA polymerase kit).

Amplification was performed on a Biometra Professional Standard Gradient thermocycler with the following conditions: (i) 94 °C for 2 min followed by 40 cycles of 94 °C for 15 s, 56 °C for 30 s, 68 °C for 5 min and a last step at 68 °C for 10 min (Platinum PCR SuperMix High Fidelity kit), (ii) 95 °C for 2 min followed by 30 cycles of 95 °C for 30 s, 57 °C for 30 s, 72 °C for 10 min and a last step at 72 °C for 10 min (Pfu DNA Polymerase, recombinant kit), or (iii) 98 °C for 2 min followed by 30 cycles of 98 °C for 10 s, 59 °C for 30 s, 72 °C for 75 s and a last step at 72 °C for 10 min (Phusion High Fidelity DNA polymerase kit).

Sizes of all DNA fragments were verified by gel electrophoresis. The Amicon Ultra 0.5 mL kit (Millipore) was used to purify PCR products. A digestion step using the restriction enzyme DpnI (New England Biolabs) was performed to ensure complete removal of the DNA template, as previously described [6].

### 2.5. Cell Transfection

The cell transfection procedure was performed as previously described [7]. Briefly, an amount of 1 µg of IC or of an equimolar mix of the three DNA fragments was used to transfect a 12.5 cm^2^ culture flask of subconfluent HEK-293 cells using the Lipofectamine 3000 reagent (Thermo Scientific). Cell supernatant was removed after an incubation of 24 h, and 3.5 mL of fresh medium was added. Cell supernatants were harvested when a cytopathic effect (CPE) was observed, 6–7 days later.

Each infectious cell supernatant was then passaged twice on Vero cells, as previously described [7]. Briefly, 333 µL of clarified cell supernatant was inoculated onto a 12.5 cm^2^ culture flask. Cell supernatants were harvested after 6–7 days. Clarified cell supernatants (virus stocks) were used to perform quantification of viral RNA yields, TCID_50_ assay, and whole-genome sequencing.

### 2.6. Quantitative Real-Time (RT)-PCR Assays

The EZ1 mini virus 2.0 kit and the EZ1 Biorobot (both from Qiagen, Hilden, Allemagne) were employed to extract viral RNA from cell supernatant according to the manufacturer’s instructions. As previously described, to assess the production of viral particles in cell supernatant, we quantified viral RNA yields using the GoTaq Probe One-Step RT-qPCR System (Promega, Madison, WI, USA), and we detected any amount of remaining DNA using the Takyon qPCR kit (Eurogentec Liège, Belgique) [7]. Primers and probe sequences are listed in Table S2. After two passages, amounts of remaining DNA were negligible compared to RNA thresholds (more than 10^6^ times lower). Viral RNA yields were assessed from standard curves. 

### 2.7. Tissue Culture Infectious Dose 50 (TCID_50_) Assay

As previously described, a 96 well plate of confluent Vero cells (100 µL of medium per well) was inoculated with 50 μL/well of serial 10-fold dilutions of clarified cell supernatants [7]. Each row contained six wells of the same dilution and two negative controls. Plates were incubated for 7 days, and absence or presence of CPE in each well was observed. Infectious titres were estimated using the method of Reed and Muench [22].

### 2.8. Sequence Analysis of the Full-Length Genome

Viral RNA extraction from cell supernatant was performed as described above. A set of specific primer pairs (Table S3) was used to produce overlapping amplicons covering the entire viral genome with the SuperScript III One-Step RT-PCR System (Thermo Scientific). Complete genome sequencing was performed using the Ion PGM Sequencer (Thermo Scientific). Read sequences were analyzed as previously described [7]. To assess the genetic diversity of viral populations, mutation frequency for each position was calculated as the number of reads with a mutation compared to the reference, divided by the total number of reads at that site (minimum coverage of 500). For the analysis, only substitutions with a frequency of at least 1% were taken into account (Table S4).

### 2.9. In Vivo Experiments

Group of three week old C57Bl/6J female mice were intra-peritoneally inoculated with 100 μL of virus. For each experiment, a control group of eight mice was used (they were intra-peritoneally inoculated with 100 μL of PBS). Following infection, mice were monitored daily for the duration of the study. The clinical course of the infection was monitored by following the weight of the mice and the appearance of symptoms (shivering, humpback, dirty eyes, hemi- or tetra-paresia, hemiplegia or tetraplegia). Mice with hemiplegia/tetraplegia, that became moribund, or that lost greater than 20% of their maximum weight were euthanized. Brains and spleens were sampled from sacrificed mice and collected in a 1.5 mL tube that contained 500 µL of Hank’s Balanced Salt Solution (Thermo Scientific) supplemented with 20% of FBS and a tungsten bead. They were grounded using a MM300 mixer (Retsch) for 4 min at 30 cycles/s, and then centrifuged 5 min at 3500 rpm. Supernatants were then stored at −80 °C. Quantitative real-time PCR assays were performed using these supernatants. Nucleic acid extraction using 40 μL of supernatant, 150 μL of AVL buffer (Qiagen), and 10 μL of MS2 bacteriophage (internal control) was accomplished using the EZ1 mini virus 2.0 kit and the EZ1 Biorobot (both from Qiagen). As described above, viral RNA yields were quantified using the GoTaq Probe One-Step RT-qPCR System (Promega). Results were normalized using amplification (qRT-PCR) of the housekeeping gene HMBS, as described previously [19].

### 2.10. Statistical Analysis

Statistical tests (Shapiro–Wilk test, Wilcoxon test, Fischer test, Welch test, and Student test) were carried out with R software [23]. Log-rank test were performed with GraphPad Prism software.

## 3. Results

An infectious clone (IC) of the TBEV strain Oshima was directly used for cell transfection. Infectious particles were rescued, and this virus was named Osh_IC. The same IC was also used as template to generate subgenomic DNA amplicons using three PCR amplification kits with different fidelities: the Platinum PCR SuperMix High Fidelity kit and the Pfu DNA Polymerase recombinant kit, considered high-fidelity kits (error rate of 3.67 × 10^−6^ and 2.6 × 10^−6^ respectively), and the Phusion High Fidelity DNA polymerase kit, considered a very high-fidelity kit (error rate of 4.4 × 10^−7^). Infectious particles were rescued following the ISA procedure. The corresponding viruses were named Osh_ISAtaq, Osh_ISApfu, and Osh_ISAphu. For all the viruses, we used HEK293 cells for transfections, and viruses were passaged twice in Vero cells before genotypic and phenotypic comparative analyses.

### 3.1. In Cellulo Experiments: Genetic Diversity and Replicative Fitness

To study the impact of the reverse genetic procedure on genetic diversity of viral populations, the complete genome sequences of Osh_IC, Osh_ISAtaq, Osh_ISApfu, and Osh_ISAphu viruses were established by NGS method (only substitutions with a frequency of at least 1% were taken into account). Results from three independent transfections were used for each condition.

As previously shown with CHIKV, viruses produced using the ISA method exhibited a higher number of mutations than Osh_IC viruses (mean values: 34, 21.7, and 17.3 vs. 2.3 respectively for Osh_ISAtaq, Osh_ISApfu, and Osh_ISAphu viruses vs. Osh_IC viruses) [17]. This difference was only significant for Osh_ISAphu viruses (Student test; *p*  =  0.0091).

**Figure 1:**
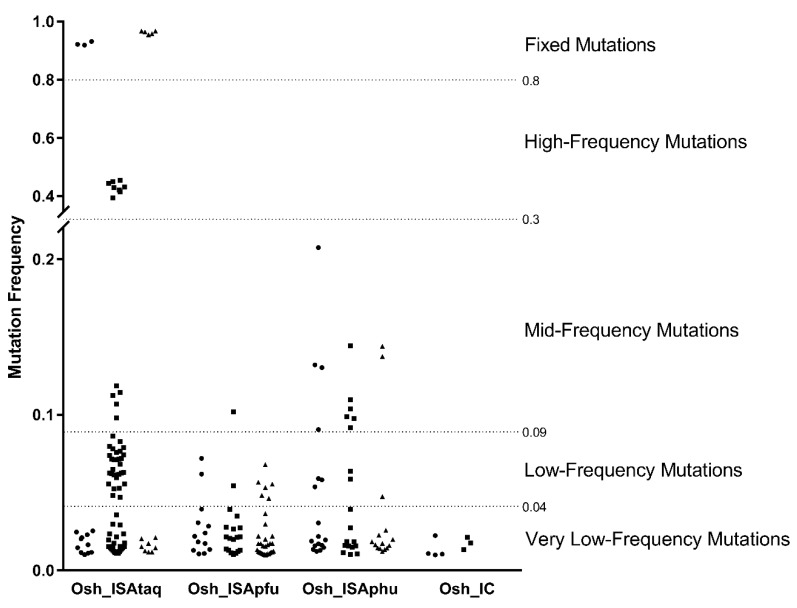
Genetic diversity of viral populations. The complete genome sequence of each virus was determined in triplicate. All substitutions detected with a frequency of at least 1% are represented by circles, squares, or triangles, which correspond to the first, second, and third replicates, respectively. Five mutation categories were defined according to frequency distribution. No mutation was detected for the third replicate of the Osh_IC virus.

Analysis of all mutations allowed us to define five type of mutations based on their frequency: very low-frequency, low-frequency, mid-frequency, high-frequency, and fixed mutations (Figure 1). As expected, only very low-frequency mutations were detected with Osh_IC viruses (one replicate did not show any mutations). Conversely, high-frequency and fixed mutations were only identified with Osh_ISAtaq viruses. Low- and mid-frequency mutations were detected with all the viruses generated using the ISA method. Of note, two different profiles were observed with Osh_ISAtaq viruses (replicates #1 and #3 versus replicate #2; Figure 1). Altogether, these results confirm our initial hypothesis: the use of a very high-fidelity PCR polymerase (i.e., Phusion DNA polymerase) during the ISA procedure decreases the genetic diversity of viral populations. However, comparative genotypic analysis also revealed that two PCR kits with similar fidelities (i.e., the Platinum PCR SuperMix High Fidelity kit and the Pfu DNA Polymerase, recombinant kit) can lead to genetically different viral populations.

**Figure 2:**
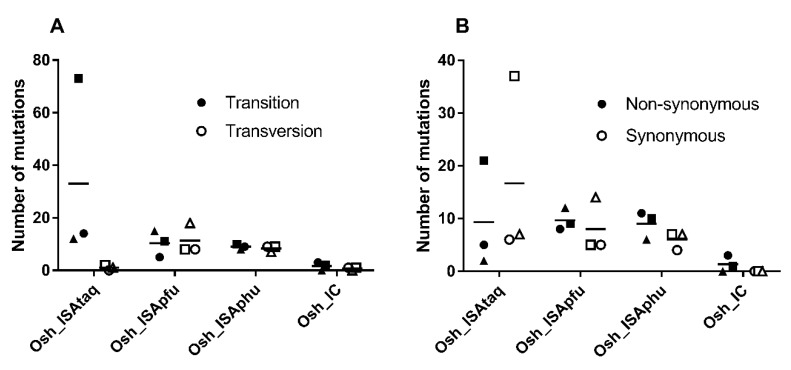
Mutation characteristics. The complete genome sequence of each virus was determined in triplicate. This figure represents the number of transition/transversion (**A**) and non-synonymous/synonymous (**B**) mutations. Circles, squares, or triangles correspond to the first, second, and third replicates, respectively, and dashes represent mean values.

We found a majority of transition with the Osh_IC and Osh_ISAtaq viruses, whereas Osh_ISApfu and Osh_ISAphu exhibited approximately the same number of transitions and transversions (Figure 2). Unlike the Osh_ISAtaq virus, a majority of non-synonymous mutations was detected with Osh_IC, Osh_ISApfu, and Osh_ISAphu. None of these differences here significant (Student, Welch, and Wilcoxon tests).

Mutations were distributed throughout the genome with all the viruses generated using the ISA method (Figure S1). Since very few mutations were detected with the Osh_IC viruses, their distribution through four genomic regions is complicated to interpret.

**Figure 3:**
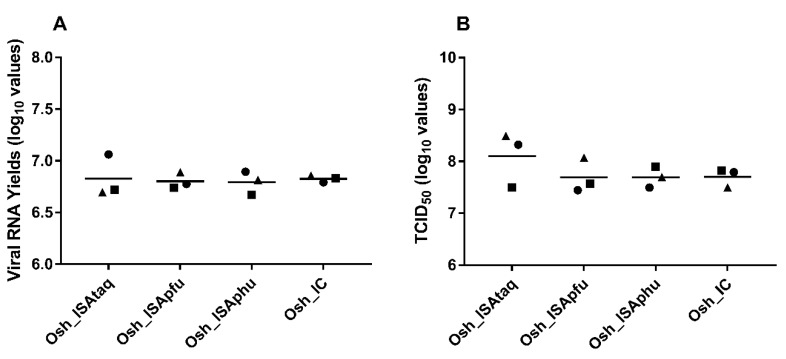
Viral replicative fitness. Viral loads in cell supernatant media of each replicate were estimated using a real-time RT-PCR assay (molecular viral loads) (**A**) and a Tissue Culture Infectious Dose 50 (TCID_50_) assay (infectious titers) (**B**). Circles, squares, and triangles correspond to the first, second, and third replicates, respectively, and dashes represent mean values.

To study the impact of the reverse genetic procedure on in cellulo replicative fitness, amounts of viral RNA (qRT-PCR assay) and infectious titres (TCID_50_ assay) in cell supernatants were established. Results from three independent transfections were used for each condition. In all cases, similar values were found and no significant difference was observed (Student and Welch tests; Figure 3).

### 3.2. In Vivo Experiments: Viral Pathogenicity

To evaluate the impact of the reverse genetic procedure on viral pathogenicity, we used a relevant mouse model (pathological changes in mouse brains as well as clinical signs are similar to those observed in humans) [18,19,20]. We worked with the Osh_IC virus, Osh_ISAtaq virus, and Osh_ISA phu virus. Because similar results were found in cellulo with the Osh_ISApfu and Osh_ISA phu viruses, we selected the latter, which exhibited, based on manufacturer instructions, the lowest error prone rate (see above).

**Figure 4:**
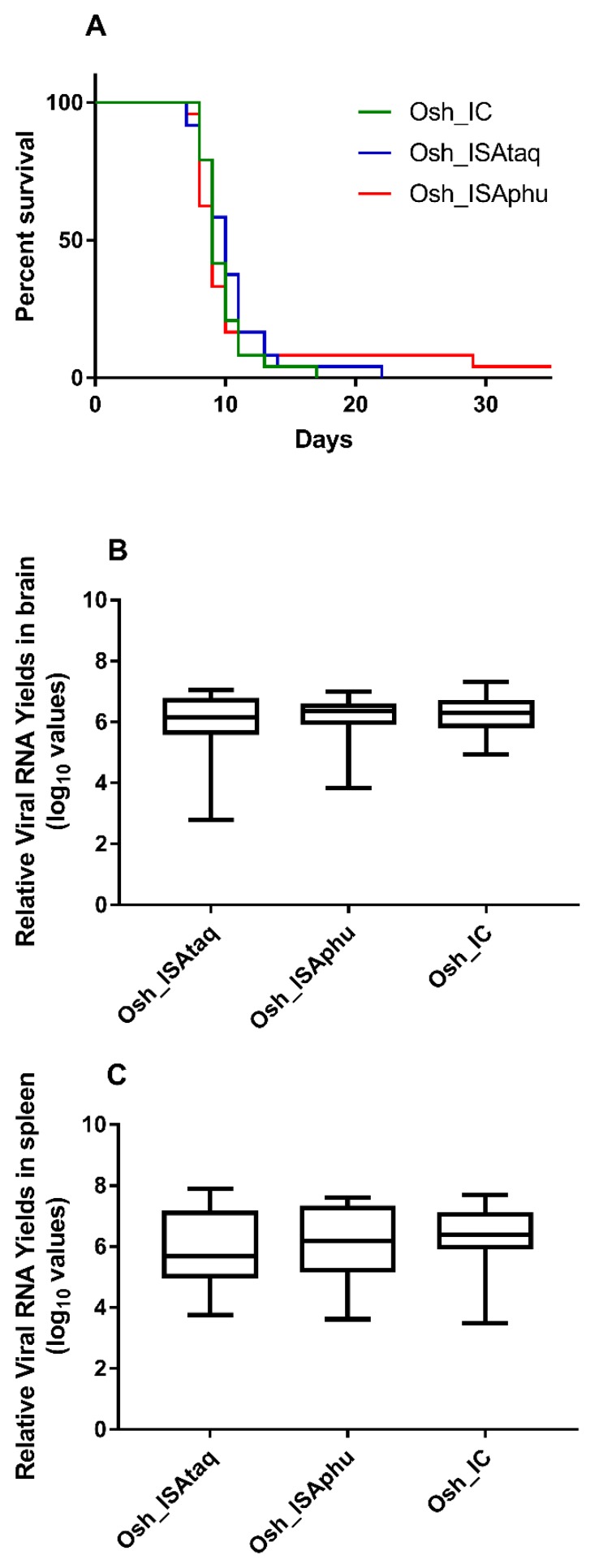
Survival curves (**A**) and viral RNA yields in collected organs (**B**–**C**). Group of twenty four mice were intra-peritoneally inoculated with 2 × 10^6^ TCID_50_ of each virus. Survival curves were established based on human endpoints (**A**). Brains (**B**) and spleens (**C**) of these animals were used to determine viral RNA yields using a quantitative real-time RT-PCR assay. The bottoms and tops of the boxes represent the first and third quartiles, the dashes inside the boxes represent the median values, and the ends of the error bars represent the minimum and maximum values.

Groups of three week old female C57Bl/6 mice were inoculated intra-peritoneally with 100 μL that contained 2 × 10^6^ TCID_50_/mL of the Osh_IC, Osh_ISAtaq, or Osh_ISAphu virus. A group of uninfected mice was used (intra-peritoneally inoculated with 100 μL of PBS) as a negative control. Mice were monitored daily to assess survival rate based on human endpoints. Viral RNA yields detected in brains and spleens of these mice were determined using a qRT-PCR assay. Using mortality as criterion, similar Kaplan–Meier curves were obtained for Osh_IC, Osh_ISAtaq, and Osh_ISAphu viruses (Log-rank test; *p* > 0.05; Figure 4): following 7 days, almost all the mice (>98%) developed a severe infection associated with high mortality. Amounts of viral RNA found in brains and in spleens were similar between the three groups of mice, and no significant difference was observed (Student and Wilcoxon tests; Figure 4). In addition, no significant difference was observed with Kaplan–Meier curves obtained using as criterion a weight loss of more than 15% of their maximum weight and appearance of at least one symptom (Figure S2).

**Figure 5:**
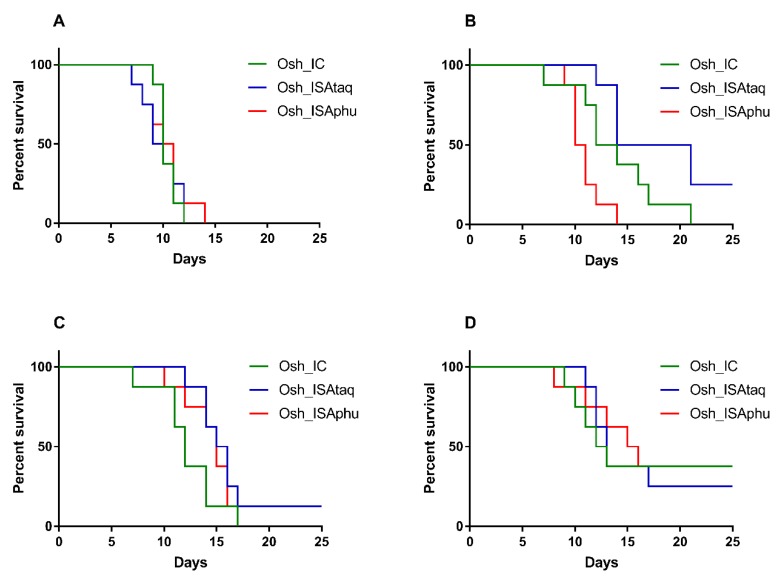
Survival curves according to the dose of virus inoculated. Group of eight mice were intra-peritoneally inoculated with 2 × 10^5^ (**A**), 2 × 10^4^ (**B**), 2 × 10^3^ (**C**), or 2 × 10^2^ (**D**) TCID_50_ of each virus. Survival curves were established based on human endpoints.

In order to confirm this result with lower doses, groups of three week old female C57Bl/6 mice were inoculated intra-peritoneally with 100 μL that contained 2 × 10^5^, 2 × 10^4^, 2 × 10^3^, or 2 × 10^2^ TCID_50_ of each virus. Similar survival curves were obtained for the Osh_IC, Osh_ISAtaq, and Osh_ISAphu viruses (Log-rank test; *p* > 0.05 except between the Osh_ISAtaq and Osh_ISAphu viruses with a dose of 2 × 10^4^ TCID_50_/mL; Figure 5). Altogether, these findings demonstrate that viral pathogenicity was not affected by the reverse genetic procedure used to generate the virus.

## 4. Discussion

The ISA method is a new reverse genetic method that circumvents some limitations related to the use of IC (e.g., cloning and propagation of cDNA copy of the complete genome into bacteria, in vitro RNA transcription). We previously demonstrated with CHIKV that use of the ISA method resulted in the production of a genetically heterogeneous viral population. Here, we confirmed this result with another virus, TBEV, which belongs to another family; transfection of PCR products generated higher genetic diversity of viral populations than transfection of an IC. As a result, following cell transfection and two passages in Vero cells, we observed with Osh_ISAtaq viruses a higher number of mutations than with Osh_IC viruses. In addition, at least three high-frequency or fixed mutations were detected with Osh_ISAtaq viruses, while none were found following transfection of an IC. It is of note that two different profiles were observed with Osh_ISAtaq viruses. It is likely that in replicate #1 and replicate #3, one variant emerged during viral replication. For replicate #2, cell supernatant probably contained two different variants.

We recently developed a new ISA-derived method, named SuPReMe (Subgenomic Plasmids Recombination Method), that gives the possibility to produce a quasi-clonal viral population. Contrary to the ISA method, SuPReMe is suitable for determining the impacts of mutations on the biological properties of viruses.

In the present study, we propose another strategy for generation of quasi-clonal viral populations. Indeed, we hypothesized that the level of genetic heterogeneity of viral populations is positively correlated with the rate of error-prone nucleotide incorporation of the thermostable DNA-dependent DNA polymerases (i.e., fidelity) used during the PCR step of the ISA procedure. To confirm this assumption, we explored the possibility of working with a very high-fidelity PCR polymerase: the Phusion High Fidelity DNA polymerase. Analysis of the genetic diversity of viral populations after two passages in Vero cells corroborated our initial hypothesis: a reduced genetic heterogeneity was observed with Osh_ISAphu viruses. However, viral populations obtained were not as genetically homogeneous as those obtained with an IC. Altogether, these results indicate that the use of a very high-fidelity PCR polymerase during the ISA procedure is a suitable alternative method for generating quasi-clonal populations of viruses.

Although the Pfu DNA Polymerase recombinant kit and Platinum PCR SuperMix High Fidelity kit amplified with similar fidelity, Osh_ISApfu viruses exhibited a reduced genetic heterogeneity compared to Osh_ISAtaq viruses. However, our results suggest that the nature of the PCR polymerase induced specific mutational patterns (i.e., transition/transversion and synonymous/non-synonymous mutations), in accordance with previous studies which analyzed in vitro DNA polymerase fidelity [10,24,25,26]. Moreover, it is likely that the genetic variability observed in the present study was also the consequence of early evolutionary events that occurred in cellulo (i.e., stochastic or adaptation mutations during viral replication). Therefore, the genetic diversity of viral populations does not depend solely on the fidelity of the polymerase used during the PCR step of the ISA method. Mutational patterns induced by the polymerases (the one used during the PCR and the viral) and early evolutionary events may also have an impact on genetic variability.

The second objective of this study was to compare in vitro and in vivo the phenotype of the viruses generated using different reverse genetic procedures. Indeed, we previously demonstrated with the Chikungunya virus that neither in vitro replicative fitness, nor vector competence in Aedes mosquitoes were affected by the reverse genetic method used (infectious clone or ISA method). Here, we confirmed that the choice of reverse genetic method used did not affect the viral phenotype, using another virus and another animal model. Indeed, our results showed similar viral replicative fitness for the Osh_IC, Osh_ISAtaq, Osh_ISApfu, and Osh_ISAphu viruses. In vivo experiments also indicated that the viral pathogenicity was not significantly impacted by the reverse genetic method used. All these results validate the use of the ISA method for in vitro and in vivo experiments, particularly for the study of viral pathogenicity.

In conclusion, in this study we have developed an ISA-based approach to control viral population diversity without modifying virus phenotype. Because the ISA method has been previously applied to a wide range of single-stranded positive-sense RNA viruses, it is likely that this new approach will be applicable to these viruses without affecting their phenotypes. In addition, the recent improvements of the ISA method could take advantage of these results [27,28].

Our results confirmed that the PCR step during the ISA method affects the genetic diversity of viral populations. However, contrary to what we initially thought, the fidelity of the polymerase is not the only factor influencing this diversity. The study of the impact of mutational characteristics of the polymerases used during the PCR step of the ISA method on the genetic diversity of viral populations could be the subject of further work.

## Supplementary Materials

The following are available online at https://www.mdpi.com/1999-4915/11/7/666/s1. Table S1: Primers sequences used for the PCR step of the ISA method. Table S2: Primers and probe sequences used for the quantitative real-time (RT)-PCR assays. Table S3: Primer sequences used to produce overlapping amplicons covering the entire viral genome, for sequencing. Table S4: Summary of all mutations detected. Figure S1: Mutations distribution. This figure summarizes the distribution of single nucleotide polymorphic sites detected on different regions of the viral genome. Figure S2: Kaplan-Meier survival analysis using as criteria, a weight loss of more than 15% (A) and appearance of at least one symptom (B).
