# Supplementary material for: Reverse Genetics of RNA Viruses: ISA-Based Approach to Control Viral Population Diversity without Modifying Virus Phenotype
Source: Viruses. 2019 Jul 20;11(7):666. doi: 10.3390/v11070666 (PMC6669666; doi:10.3390/v11070666)
Supplement: Supplementary file 1 [file viruses-11-00666-s001.zip › Figure S1.pdf]

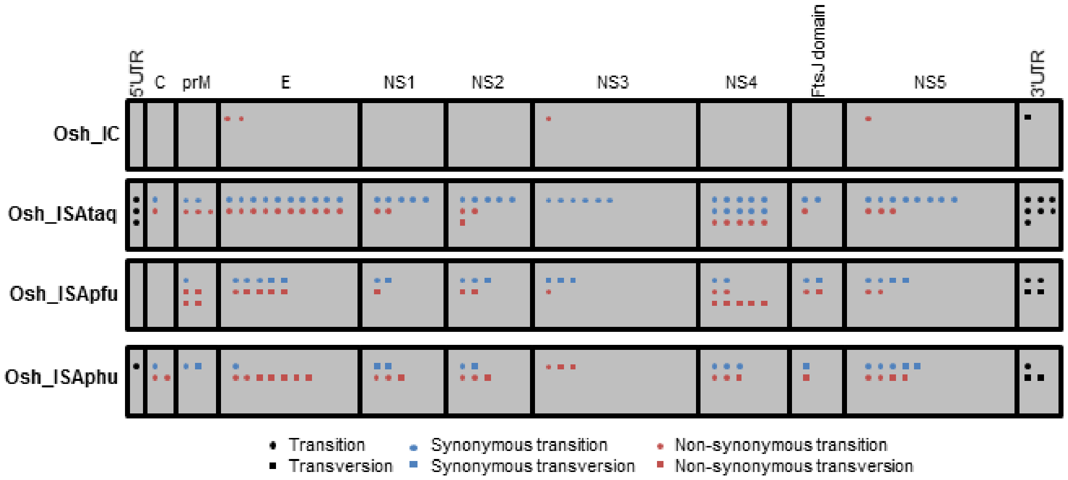

Figure S1: Mutations distribution. This figure summarizes the distribution of single nucleotide polymorphic sites detected on different regions of the viral genome.
