# Supplementary material for: Reverse Genetics of RNA Viruses: ISA-Based Approach to Control Viral Population Diversity without Modifying Virus Phenotype
Source: Viruses. 2019 Jul 20;11(7):666. doi: 10.3390/v11070666 (PMC6669666; doi:10.3390/v11070666)
Supplement: Supplementary file 1 [file viruses-11-00666-s001.zip › Table S1.pdf]

	Forward	Reverse
First Fragment	CAGGGTTATTGTCTCATGAGCGGA	GCCACGCCCAGGAAGAGCATGA
Second Fragment	GGGCCCTCTGGAAATGGGGAGA	CAACCCAGGCTTGTCACCATCTTT
Third Fragment	GGGTGAGGTCGTGGACCTTGGA	CCTAGGAATTTCACAAATAAAGCATTTT

Table S1: Primers sequences used for the PCR step of the ISA method.
