# Supplementary material for: Reverse Genetics of RNA Viruses: ISA-Based Approach to Control Viral Population Diversity without Modifying Virus Phenotype
Source: Viruses. 2019 Jul 20;11(7):666. doi: 10.3390/v11070666 (PMC6669666; doi:10.3390/v11070666)
Supplement: Supplementary file 1 [file viruses-11-00666-s001.zip › Table S2.pdf]

Forward Primer	GCAGAGTGGGCCAGGAACAT
Probe	TCGGACAAGAGAAGTTCAAGGACT
Reverse Primer	GTCATGCCGATCCATGCAGGA

Table S2: Primers and probe sequences used for the quantitative real-time (RT)-PCR assays.
