# Supplementary material for: Reverse Genetics of RNA Viruses: ISA-Based Approach to Control Viral Population Diversity without Modifying Virus Phenotype
Source: Viruses. 2019 Jul 20;11(7):666. doi: 10.3390/v11070666 (PMC6669666; doi:10.3390/v11070666)
Supplement: Supplementary file 1 [file viruses-11-00666-s001.zip › Table S3.pdf]

	Forward	Reverse
First Fragment	AGATTTTCTTGCACGTGCATGCGT	GTGTTATCAACATAGCCACGTTCA
Second Fragment	ACAGTGGACATGATACAGTGGTCA	GCCACTAACTGCATCTTCCTTGT
Third Fragment	CATGGAAGGAGACGGTCATTCAGT	CCTCTCGGCCATCTTCATTGCTCT
Fourth Fragment	GGATGCGCGCATGTTCAGAGA	TGCCAGTCGTTCCAGAATCCAGT
Fifth Fragment	AGGATGTCGTGCGAATGGCCAT	AGCGGGTGTTTTTCCGAGTCACTCA

Table S3: Primer sequences used to produce overlapping amplicons covering the entire viral genome, for sequencing.
