# Supplementary material for: Reverse Genetics of RNA Viruses: ISA-Based Approach to Control Viral Population Diversity without Modifying Virus Phenotype
Source: Viruses. 2019 Jul 20;11(7):666. doi: 10.3390/v11070666 (PMC6669666; doi:10.3390/v11070666)
Supplement: Supplementary file 1 [file viruses-11-00666-s001.zip › Table S4.pdf]

Virus	Mutation Position	Region	Frequency	Nucleotide	Transition / Transversion	Amino acid	Synonymous / Non-Synonymous
Osh_IC #1	2347	glycoprot	0,0100	A->G	Transition	Asp -> Gly	Non-Synonymous
Osh_IC #1	6147	DEXDc	0,0105	G->A	Transition	Ala -> Thr	Non-Synonymous
Osh_IC #1	10503	NS5	0,0108	G->A	Transition	Asp -> Asn	Non-Synonymous
Osh_IC #1	11859	3' UTR	0,0224	A->C	Transversion		UTR
Osh_IC #2	8528		0,0135	C->T	Transition		
Osh_IC #2	2511	glycoprot	0,0176	G->A	Transition	Gly -> Arg	Non-Synonymous
Osh_IC #2	11859	3' UTR	0,0214	A->C	Transversion		UTR
Osh_ISAtaq #1	2833	E	0,0101	A->G	Transition	Glu -> Gly	Non-Synonymous
Osh_ISAtaq #1	7427	NS4A	0,0111	C->T	Transition	Gly	Synonymous
Osh_ISAtaq #1	826	5'UTR	0,0116	A->G	Transition		UTR
Osh_ISAtaq #1	11628	3' UTR	0,0117	T->C	Transition		UTR
Osh_ISAtaq #1	6020	DEXDc	0,0146	T->C	Transition	Ile	Synonymous
Osh_ISAtaq #1	8216	NS4B	0,0165	T->C	Transition	Leu	Synonymous
Osh_ISAtaq #1	2362	glycoprot	0,0204	A->G	Transition	His -> Arg	Non-Synonymous
Osh_ISAtaq #1	2195	glycoprot	0,0212	T->C	Transition	Ala	Synonymous
Osh_ISAtaq #1	1641	M	0,0229	A->G	Transition	Thr -> Ala	Non-Synonymous
Osh_ISAtaq #1	1627	M	0,0248	A->G	Transition	Lys -> Arg	Non-Synonymous
Osh_ISAtaq #1	4715	NS2A	0,0254	T->C	Transition	Ala	Synonymous
Osh_ISAtaq #1	2504	glycoprot	0,9193	C->T	Transition	Tyr	Synonymous
Osh_ISAtaq #1	11385	3' UTR	0,9221	G->A	Transition		UTR
Osh_ISAtaq #1	7324	NS4A	0,9317	A->G	Transition	His -> Arg	Non-Synonymous
Osh_ISAtaq #2	1799	glycoprot	0,0110	A->G	Transition	Arg	Synonymous
Osh_ISAtaq #2	4542	NS2A	0,0110	T->C	Transition	Leu	Synonymous
Osh_ISAtaq #2	11279	3' UTR	0,0112	A->G	Transition		UTR
Osh_ISAtaq #2	3359	NS1	0,0112	A->G	Transition	Ser	Synonymous
Osh_ISAtaq #2	7932	NS4B	0,0116	C->T	Transition	Pro -> Ser	Non-Synonymous
Osh_ISAtaq #2	8615	FGsJ	0,0120	A->G	Transition	Lys	Synonymous
Osh_ISAtaq #2	2270	glycoprot	0,0121	A->G	Transition	Gly	Synonymous
Osh_ISAtaq #2	8892	FGsJ	0,0122	G->A	Transition	Ala -> Thr	Non-Synonymous
Osh_ISAtaq #2	6554	HELICc	0,0123	T->C	Transition	Phe	Synonymous
Osh_ISAtaq #2	9268	NS5	0,0129	A->G	Transition	Glu -> Arg	Non-Synonymous
Osh_ISAtaq #2	6758	HELICc	0,0131	C->T	Transition	Val	Synonymous
Osh_ISAtaq #2	8066	NS4B	0,0138	T->C	Transition	Ala	Synonymous
Osh_ISAtaq #2	1247		0,0139	A->G	Transition		
Osh_ISAtaq #2	834	5'UTR	0,0144	A->G	Transition		UTR
Osh_ISAtaq #2	4874	NS2A	0,0144	A->G	Transition	Gly	Synonymous
Osh_ISAtaq #2	4904	NS2A	0,0145	A->G	Transition	Leu	Synonymous
Osh_ISAtaq #2	7731	NS4B	0,0148	A->G	Transition	Ser -> Gly	Non-Synonymous
Osh_ISAtaq #2	2240	glycoprot	0,0149	T->C	Transition	Val	Synonymous

Osh_ISAtaq #2	8509		0,0150	C->T	Transition		
Osh_ISAtaq #2	2131	glycoprot	0,0152	T->C	Transition	Val -> Ala	Non-Synonymous
Osh_ISAtaq #2	2972	E	0,0154	A->G	Transition	Glu	Synonymous
Osh_ISAtaq #2	9833	NS5	0,0155	A->G	Transition	Gly	Synonymous
Osh_ISAtaq #2	8048	NS4B	0,0155	G->A	Transition	Val	Synonymous
Osh_ISAtaq #2	4360	NS2A	0,0156	G->T	Transversion	Arg -> Leu	Non-Synonymous
Osh_ISAtaq #2	819	5'UTR	0,0174	A->G	Transition		UTR
Osh_ISAtaq #2	6350	DEXDc	0,0176	T->C	Transition	Ser	Synonymous
Osh_ISAtaq #2	8528		0,0196	C->T	Transition		
Osh_ISAtaq #2	7447	NS4A	0,0217	T->C	Transition	Val -> Ala	Non-Synonymous
Osh_ISAtaq #2	6404		0,0235	T->C	Transition		
Osh_ISAtaq #2	8548		0,0236	A->G	Transition		
Osh_ISAtaq #2	3177	E	0,0292	T->C	Transition	Phe -> Leu	Non-Synonymous
Osh_ISAtaq #2	4052	NS1	0,0296	A->G	Transition	Arg	Synonymous
Osh_ISAtaq #2	4922	NS2A	0,0356	T->C	Transition	Ser	Synonymous
Osh_ISAtaq #2	8057	NS4B	0,0468	A->G	Transition	Gly	Synonymous
Osh_ISAtaq #2	9280	NS5	0,0480	G->A	Transition	Ser -> Asn	Non-Synonymous
Osh_ISAtaq #2	9542	NS5	0,0524	C->T	Transition	Gly	Synonymous
Osh_ISAtaq #2	1782	glycoprot	0,0527	A->G	Transition	Thr -> Ala	Non-Synonymous
Osh_ISAtaq #2	1577	M	0,0553	A->G	Transition	Glu	Synonymous
Osh_ISAtaq #2	9922	NS5	0,0556	A->G	Transition	His -> Arg	Non-Synonymous
Osh_ISAtaq #2	10961	NS5	0,0596	T->C	Transition	Ser	Synonymous
Osh_ISAtaq #2	8224	NS4B	0,0614	T->C	Transition	Met -> Thr	Non-Synonymous
Osh_ISAtaq #2	1382	propep	0,0616	A->G	Transition	Ser	Synonymous
Osh_ISAtaq #2	3212	E	0,0622	A->G	Transition	Thr	Synonymous
Osh_ISAtaq #2	10340	NS5	0,0625	A->G	Transition	Ala	Synonymous
Osh_ISAtaq #2	3435	NS1	0,0626	A->G	Transition	Ser -> Gly	Non-Synonymous
Osh_ISAtaq #2	10421	NS5	0,0628	C->T	Transition	Gly	Synonymous
Osh_ISAtaq #2	8441		0,0650	T->C	Transition		
Osh_ISAtaq #2	2251	glycoprot	0,0682	A->G	Transition	Lys -> Arg	Non-Synonymous
Osh_ISAtaq #2	2300	glycoprot	0,0710	A->G	Transition	Arg	Synonymous
Osh_ISAtaq #2	10553	NS5	0,0711	C->T	Transition	Val	Synonymous
Osh_ISAtaq #2	2047	glycoprot	0,0714	A->G	Transition	Asn -> Ser	Non-Synonymous
Osh_ISAtaq #2	7595	NS4A	0,0716	G->A	Transition	Ala	Synonymous
Osh_ISAtaq #2	1079	C prot	0,0719	G->A	Transition	Lys	Synonymous
Osh_ISAtaq #2	9285	NS5	0,0738	T->C	Transition	Leu	Synonymous
Osh_ISAtaq #2	6425		0,0742	A->G	Transition		
Osh_ISAtaq #2	8705	FGsJ	0,0759	A->G	Transition	Ala	Synonymous
Osh_ISAtaq #2	5861	NS3	0,0766	C->T	Transition	Ser	Synonymous
Osh_ISAtaq #2	4734	NS2A	0,0780	A->G	Transition	Thr -> Ala	Non-Synonymous
Osh_ISAtaq #2	8579		0,0788	C->T	Transition		
Osh_ISAtaq #2	3302		0,0797	A->G	Transition		
Osh_ISAtaq #2	7262	NS4A	0,0829	G->A	Transition	Gly	Synonymous

Osh_ISAtaq #2	3848	NS1	0,0862	T->C	Transition	Asn	Synonymous
Osh_ISAtaq #2	991	C prot	0,0979	T->C	Transition	Val -> Ala	Non-Synonymous
Osh_ISAtaq #2	11209	3' UTR	0,1067	A->G	Transition		UTR
Osh_ISAtaq #2	1462	propep	0,1122	T->C	Transition	Val -> Ala	Non-Synonymous
Osh_ISAtaq #2	11746	3' UTR	0,1143	C->T	Transition		UTR
Osh_ISAtaq #2	11714	3' UTR	0,1185	A->G	Transition		UTR
Osh_ISAtaq #2	3372	NS1	0,3948	A->G	Transition	Thr -> Ala	Non-Synonymous
Osh_ISAtaq #2	11716	3' UTR	0,4156	A->G	Transition		UTR
Osh_ISAtaq #2	7520	NS4A	0,4206	T->C	Transition	Gly	Synonymous
Osh_ISAtaq #2	2509	glycoprot	0,4299	T->A	Transversion	Leu -> His	Non-Synonymous
Osh_ISAtaq #2	1237		0,4325	T->C	Transition		
Osh_ISAtaq #2	2139	glycoprot	0,4432	G->A	Transition	Ala -> Thr	Non-Synonymous
Osh_ISAtaq #2	2111	glycoprot	0,4496	A->G	Transition	Lys	Synonymous
Osh_ISAtaq #2	3674	NS1	0,4544	A->G	Transition	Arg	Synonymous
Osh_ISAtaq #3	7409	NS4A	0,0117	C->T	Transition	Leu	Synonymous
Osh_ISAtaq #3	2948	E	0,0119	T->C	Transition	Phe	Synonymous
Osh_ISAtaq #3	8528		0,0125	C->T	Transition		
Osh_ISAtaq #3	2555	glycoprot	0,0145	T->A	Transversion	Pro	Synonymous
Osh_ISAtaq #3	2356	glycoprot	0,0152	C->T	Transition	Ser -> Leu	Non-Synonymous
Osh_ISAtaq #3	10124	NS5	0,0174	A->G	Transition	Gln	Synonymous
Osh_ISAtaq #3	6045	DEXDc	0,0204	T->C	Transition	Leu	Synonymous
Osh_ISAtaq #3	6827		0,0211	T->C	Transition		
Osh_ISAtaq #3	7835	NS4B	0,9545	A->G	Transition	Pro	Synonymous
Osh_ISAtaq #3	1687		0,9580	T->C	Transition		
Osh_ISAtaq #3	6397		0,9647	A->G	Transition		
Osh_ISAtaq #3	2128	glycoprot	0,9675	A->G	Transition	His -> Arg	Non-Synonymous
Osh_ISAtaq #3	3482	NS1	0,9678	T->C	Transition	Asn	Synonymous
Osh_ISApfu #1	1668	M	0,0106	C->A	Transversion	Leu -> Met	Non-Synonymous
Osh_ISApfu #1	2203	glycoprot	0,0109	A->G	Transition	Glu -> Gly	Non-Synonymous
Osh_ISApfu #1	11015	NS5	0,0129	C->T	Transition	Ala	Synonymous
Osh_ISApfu #1	7729	NS4B	0,0136	G->T	Transversion	Trp -> Leu	Non-Synonymous
Osh_ISApfu #1	4530	NS2A	0,0174	G->T	Transversion	Ala -> Ser	Non-Synonymous
Osh_ISApfu #1	8251	NS4B	0,0184	C->T	Transition	Pro -> Leu	Non-Synonymous
Osh_ISApfu #1	3854	NS1	0,0220	A->G	Transition	Thr	Synonymous
Osh_ISApfu #1	9395	NS5	0,0241	G->T	Transversion	Leu	Synonymous
Osh_ISApfu #1	8718	FGsJ	0,0285	G->T	Transversion	Ala -> Ser	Non-Synonymous
Osh_ISApfu #1	2486	glycoprot	0,0306	T->A	Transversion	Ala	Synonymous
Osh_ISApfu #1	4659	NS2A	0,0392	G->T	Transversion	Gly -> Trp	Non-Synonymous
Osh_ISApfu #1	1765	glycoprot	0,0618	G->T	Transversion	Arg -> Met	Non-Synonymous
Osh_ISApfu #1	8429	NS4B	0,0718	G->A	Transition	Arg	Synonymous
Osh_ISApfu #2	10912	NS5	0,0101	G->A	Transition	Gly -> Asp	Non-Synonymous
Osh_ISApfu #2	8918	FGsJ	0,0110	G->T	Transversion	Arg	Synonymous
Osh_ISApfu #2	11383	3' UTR	0,0112	G->T	Transversion		UTR

Osh_ISApfu #2	7932	NS4B	0,0121	C->T	Transition	Pro -> Ser	Non-Synonymous
Osh_ISApfu #2	1765	glycoprot	0,0129	G->T	Transversion	Arg -> Met	Non-Synonymous
Osh_ISApfu #2	11682	3' UTR	0,0130	C->T	Transition		UTR
Osh_ISApfu #2	8528		0,0137	C->T	Transition		
Osh_ISApfu #2	8378	NS4B	0,0199	G->T	Transversion	Trp -> Cys	Non-Synonymous
Osh_ISApfu #2	4319		0,0207	C->T	Transition		
Osh_ISApfu #2	2770	E	0,0214	C->T	Transition	Ala -> Val	Non-Synonymous
Osh_ISApfu #2	1350	propep	0,0215	G->T	Transversion	Ala -> Ser	Non-Synonymous
Osh_ISApfu #2	8305	NS4B	0,0217	G->T	Transversion	Arg -> Ile	Non-Synonymous
Osh_ISApfu #2	6147	DEXDc	0,0266	G->A	Transition	Ala -> Thr	Non-Synonymous
Osh_ISApfu #2	8773	FGsJ	0,0274	A->G	Transition	Lys -> Arg	Non-Synonymous
Osh_ISApfu #2	7150		0,0277	G->A	Transition		
Osh_ISApfu #2	11099	NS5	0,0350	G->A	Transition	Arg	Synonymous
Osh_ISApfu #2	6275	DEXDc	0,0390	G->T	Transversion	Arg	Synonymous
Osh_ISApfu #2	5036	NS2B	0,0544	G->A	Transition	Arg	Synonymous
Osh_ISApfu #2	3512	NS1	0,1018	C->A	Transversion	Pro	Synonymous
Osh_ISApfu #3	5711	NS3	0,0100	C->A	Transversion	Pro	Synonymous
Osh_ISApfu #3	11087	NS5	0,0102	C->A	Transversion	Ser	Synonymous
Osh_ISApfu #3	11556	3' UTR	0,0104	C->T	Transition		UTR
Osh_ISApfu #3	5195	NS2B	0,0105	G->T	Transversion	Arg	Synonymous
Osh_ISApfu #3	1976	glycoprot	0,0105	T->A	Transversion	Pro	Synonymous
Osh_ISApfu #3	8019	NS4B	0,0106	G->T	Transversion	Gly -> Cys	Non-Synonymous
Osh_ISApfu #3	6986		0,0110	T->C	Transition		
Osh_ISApfu #3	3713	NS1	0,0113	C->A	Transversion	Gly	Synonymous
Osh_ISApfu #3	1407	propep	0,0115	G->C	Transversion	Asp -> His	Non-Synonymous
Osh_ISApfu #3	6884		0,0117	T->C	Transition		
Osh_ISApfu #3	7880	NS4B	0,0117	C->T	Transition	Asn	Synonymous
Osh_ISApfu #3	1414	propep	0,0118	G->T	Transversion	Gly -> Val	Non-Synonymous
Osh_ISApfu #3	4346	NS2A	0,0122	T->C	Transition	Leu	Synonymous
Osh_ISApfu #3	4082	NS1	0,0123	G->T	Transversion	Arg -> Ser	Non-Synonymous
Osh_ISApfu #3	2087	glycoprot	0,0132	T->C	Transition	Cys	Synonymous
Osh_ISApfu #3	2406	glycoprot	0,0155	C->T	Transition	Leu	Synonymous
Osh_ISApfu #3	3132	E	0,0158	G->T	Transversion	Ala -> Ser	Non-Synonymous
Osh_ISApfu #3	1343	propep	0,0165	G->A	Transition	Val	Synonymous
Osh_ISApfu #3	9778	NS5	0,0172	C->T	Transition	Ala -> Val	Non-Synonymous
Osh_ISApfu #3	2174	glycoprot	0,0172	G->A	Transition	Pro	Synonymous
Osh_ISApfu #3	6641	HELICc	0,0172	G->T	Transversion	Val	Synonymous
Osh_ISApfu #3	1822	glycoprot	0,0173	G->T	Transversion	Gly -> Ala	Non-Synonymous
Osh_ISApfu #3	8918	FGsJ	0,0201	G->A	Transition	Arg	Synonymous
Osh_ISApfu #3	7932	NS4B	0,0221	C->T	Transition	Pro -> Ser	Non-Synonymous
Osh_ISApfu #3	8528		0,0221	C->T	Transition		
Osh_ISApfu #3	2347	glycoprot	0,0298	A->G	Transition	Asp -> Gly	Non-Synonymous
Osh_ISApfu #3	8540		0,0365	A->C	Transversion		

Osh_ISApfu #3	7383	NS4A	0,0461	T->A	Transversion	Leu -> Met	Non-Synonymous
Osh_ISApfu #3	11859	3' UTR	0,0481	A->C	Transversion		UTR
Osh_ISApfu #3	3193	E	0,0532	G->T	Transversion	Gly -> Val	Non-Synonymous
Osh_ISApfu #3	6722	HELICc	0,0554	G->T	Transversion	Val	Synonymous
Osh_ISApfu #3	7672		0,0566	C->T	Transition		
Osh_ISApfu #3	1350	propep	0,0680	G->T	Transversion	Ala -> Ser	Non-Synonymous
Osh_ISAphu #1	4407	NS2A	0,0123	G->T	Transversion	Val -> Phe	Non-Synonymous
Osh_ISAphu #1	8304	NS4B	0,0129	A->G	Transition	Arg -> Gly	Non-Synonymous
Osh_ISAphu #1	3348	NS1	0,0135	G->T	Transversion	Ala -> Ser	Non-Synonymous
Osh_ISAphu #1	11859	3' UTR	0,0147	A->C	Transversion		UTR
Osh_ISAphu #1	10869	NS5	0,0158	G->A	Transition	Val -> Ile	Non-Synonymous
Osh_ISAphu #1	5891		0,0163	C->T	Transition		
Osh_ISAphu #1	978	C prot	0,0171	C->T	Transition	Arg -> Cys	Non-Synonymous
Osh_ISAphu #1	2950	E	0,0188	A->G	Transition	Gln -> Arg	Non-Synonymous
Osh_ISAphu #1	2322	glycoprot	0,0196	G->T	Transversion	Ala -> Ser	Non-Synonymous
Osh_ISAphu #1	9872	NS5	0,0218	G->T	Transversion	Leu	Synonymous
Osh_ISAphu #1	11558	3' UTR	0,0305	C->A	Transversion		UTR
Osh_ISAphu #1	10742	NS5	0,0536	G->T	Transversion	Arg	Synonymous
Osh_ISAphu #1	2347	glycoprot	0,0581	A->G	Transition	Asp -> Gly	Non-Synonymous
Osh_ISAphu #1	935	C prot	0,0590	C->T	Transition	Gly	Synonymous
Osh_ISAphu #1	6127	DEXDc	0,0904	G->T	Transversion	Gly -> Val	Non-Synonymous
Osh_ISAphu #1	7988	NS4B	0,1302	C->T	Transition	Gly	Synonymous
Osh_ISAphu #1	5061	NS2B	0,1321	G->A	Transition	Ala -> Thr	Non-Synonymous
Osh_ISAphu #1	8917	FGsJ	0,2075	G->T	Transversion	Arg -> Leu	Non-Synonymous
Osh_ISAphu #2	3987	NS1	0,0103	T->C	Transition	Trp -> Arg	Non-Synonymous
Osh_ISAphu #2	1090	C prot	0,0106	A->G	Transition	Asn -> Ser	Non-Synonymous
Osh_ISAphu #2	10452	NS5	0,0115	A->G	Transition	Arg -> Gly	Non-Synonymous
Osh_ISAphu #2	8312	NS4B	0,0150	G->T	Transversion	Glu -> Asp	Non-Synonymous
Osh_ISAphu #2	9291	NS5	0,0158	G->A	Transition	Glu -> Lys	Non-Synonymous
Osh_ISAphu #2	1424	propep	0,0159	G->T	Transversion	Pro	Synonymous
Osh_ISAphu #2	9056	FGsJ	0,0159	G->T	Transversion	Val	Synonymous
Osh_ISAphu #2	7313	NS4A	0,0184	C->T	Transition	Tyr	Synonymous
Osh_ISAphu #2	7532	NS4A	0,0187	C->T	Transition	Tyr	Synonymous
Osh_ISAphu #2	2373	glycoprot	0,0275	G->T	Transversion	Ala -> Ser	Non-Synonymous
Osh_ISAphu #2	7126		0,0393	C->A	Transversion		
Osh_ISAphu #2	6965		0,0584	G->A	Transition		
Osh_ISAphu #2	5357	NS2B	0,0637	G->T	Transversion	Thr	Synonymous
Osh_ISAphu #2	8151	NS4B	0,0916	G->A	Transition	Gly -> Arg	Non-Synonymous
Osh_ISAphu #2	6127	DEXDc	0,0974	G->A	Transition	Gly -> Glu	Non-Synonymous
Osh_ISAphu #2	5450	NS3	0,0986	C->A	Transversion	Phe -> Leu	Non-Synonymous
Osh_ISAphu #2	9395	NS5	0,1036	G->A	Transition	Leu	Synonymous
Osh_ISAphu #2	3404	NS1	0,1098	C->A	Transversion	Pro	Synonymous
Osh_ISAphu #2	10911	NS5	0,1443	G->T	Transversion	Gly -> Cys	Non-Synonymous

Osh_ISAphu #3	3386	NS1	0,0122	A->T	Transversion	Gly	Synonymous
Osh_ISAphu #3	3803	NS1	0,0134	G->A	Transition	Met -> Ile	Non-Synonymous
Osh_ISAphu #3	3217	E	0,0140	G->T	Transversion	Gly -> Val	Non-Synonymous
Osh_ISAphu #3	866	5'UTR	0,0143	A->G	Transition		UTR
Osh_ISAphu #3	7988	NS4B	0,0145	C->A	Transversion	Gly	Synonymous
Osh_ISAphu #3	1820	glycoprot	0,0160	G->A	Transition	Leu	Synonymous
Osh_ISAphu #3	4962	NS2A	0,0165	G->A	Transition	Gly -> Arg	Non-Synonymous
Osh_ISAphu #3	1822	glycoprot	0,0174	G->T	Transversion	Gly -> Ala	Non-Synonymous
Osh_ISAphu #3	9956	NS5	0,0183	G->A	Transition	Val	Synonymous
Osh_ISAphu #3	9732	NS5	0,0199	C->A	Transversion	Gln -> Lys	Non-Synonymous
Osh_ISAphu #3	2378	glycoprot	0,0227	G->T	Transversion	Trp -> Cys	Non-Synonymous
Osh_ISAphu #3	10808	NS5	0,0258	C->A	Transversion	Gly	Synonymous
Osh_ISAphu #3	11442	3' UTR	0,0473	C->T	Transition		UTR
Osh_ISAphu #3	4937	NS2A	0,1375	G->A	Transition	Leu	Synonymous
Osh_ISAphu #3	1553	M	0,1441	A->G	Transition	Thr	Synonymous

Table S4: Summary of all mutations detected
