# Supplementary figures and images for: Reverse Genetics of RNA Viruses: ISA-Based Approach to Control Viral Population Diversity without Modifying Virus Phenotype
Source: Viruses. 2019 Jul 20;11(7):666. doi: 10.3390/v11070666 (PMC6669666; doi:10.3390/v11070666)

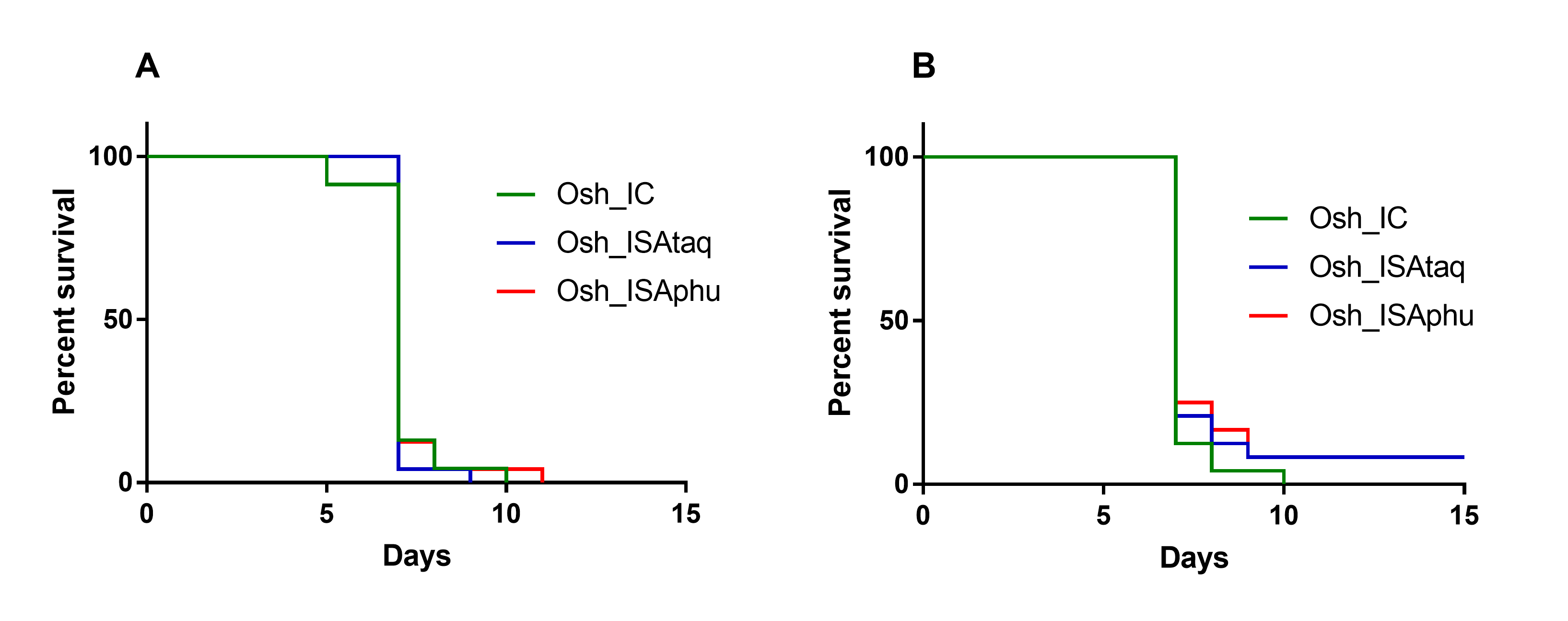

Supplement: Supplementary file 1 [file viruses-11-00666-s001.zip › Figure S2.tif]
